# The effect of phosphatidylserine on behavioral problems in children with attention deficit hyperactivity disorder

**DOI:** 10.3389/fpsyt.2026.1661725

**Published:** 2026-03-25

**Authors:** ZePing Shen, SiFan Jia, Xin Li, XiaoHong Jiang, Hang Zhang, YanTong Fang

**Affiliations:** 1 Centre for Cognition and Brain Disorders, The Affiliated Hospital, Hangzhou Normal University, Hangzhou, China; 2 Department of Neurology, The Affiliated Hospital of Hangzhou Normal University, Hangzhou, China

**Keywords:** attention-deficit hyperactivity disorder, externalizing behavior, internalizing behavior, non-pharmacological intervention, phosphatidylserine

## Abstract

Attention Deficit Hyperactivity Disorder (ADHD) is a common neurodevelopmental disorder in children, characterized by attention deficit, hyperactivity, and various internalizing and externalizing behavioral problems. Phosphatidylserine (PS), a nutritional supplement, has shown potential in improving behavioral symptoms. This study aimed to evaluate the effects of PS on core symptoms and associated behavioral problems in children with ADHD. We conducted a randomized, open-label, controlled trial involving 56 children with ADHD. Participants were randomly allocated to receive either phosphatidylserine or atomoxetine for three months. Core symptoms as well as internalizing and externalizing behaviors were assessed using the SNAP-IV rating scale and the Achenbach Child Behavior Checklist (CBCL). The PS group showed no significant improvement in core ADHD symptoms (attention deficit: M = 0.26, SD = 0.60, p > 0.05, d = 0.43; hyperactivity/impulsivity: M = 0.11, SD = 0.46, p > 0.05, d = 0.23; total score: M = 3.82, SD = 10.27, p > 0.05, d = 0.37), whereas the atomoxetine group showed significant improvements in attention deficit (M = 0.40, SD = 0.50, p < 0.05, d = 0.80), hyperactivity/impulsivity (M = 0.26, SD = 0.49, p < 0.05, d = 0.54), and total scores (M = 7.00, SD = 9.38, p < 0.05, d = 0.75). In contrast, the PS group demonstrated significant reductions in internalizing (M = 1.83, SD = 3.28, p < 0.05, d = 0.56) and externalizing behaviors (M = 3.38, SD = 5.20, p < 0.05, d = 0.65), whereas the atomoxetine group improved only in externalizing behaviors (M = 2.54, SD = 5.42, p < 0.05, d = 0.47). Further analysis revealed that both groups showed significant decreases in aggressive behaviors (PS: M = 2.79, SD = 4.24, p < 0.05, d = 0.66; atomoxetine: M = 2.43, SD = 4.90, p < 0.05, d = 0.50). Phosphatidylserine was not associated with significant improvements in core ADHD symptoms but was associated with reductions in internalizing and externalizing behavioral problems, particularly in reducing aggression. These findings suggest that PS warrants further investigation as a promising adjunctive treatment targeting behavioral dysregulation in children with ADHD.

## 1 Introduction

Attention Deficit Hyperactivity Disorder (ADHD) is a common neurodevelopmental disorder in children and adolescents, characterized by age-inappropriate inattention, hyperactivity, and impulsivity (1). In addition to these core symptoms, children with ADHD often present with internalizing and externalizing behavioral problems. Internalizing behaviors refer to emotional distress, such as anxiety, depression, and somatic symptoms. Externalizing behaviors involve noncompliance, aggression, emotional dysregulation, and hyperactivity, which affect social adaptation. Studies suggest that these behaviors not only impair the social functioning of children with ADHD but may also have long-term negative impacts on emotional and psychological health. (2, 3).

Pharmacological treatments are the first-line therapy for ADHD, primarily including stimulant and non-stimulant medications such as methylphenidate and atomoxetine (4–6). Among them, atomoxetine, a selective norepinephrine reuptake inhibitor, enhances norepinephrine and dopamine levels in the prefrontal cortex. Compared with stimulant medications, atomoxetine has a lower risk of dependence during long-term use (7). In addition to improving core symptoms, it has been shown to alleviate emotional and behavioral dysregulation in children with ADHD (8, 9). Nevertheless, its therapeutic effects on internalizing and externalizing problems are modest, and some children exhibit limited improvement or adverse effects during prolonged treatment (10). Therefore, safer complementary treatment options are needed to effectively supplement pharmacological treatments for ADHD.

Phosphatidylserine (PS) is a nutritional supplement primarily concentrated in brain cells and is involved in membrane signaling and synaptic refinement (11, 12). Studies have shown that PS supplementation significantly improves symptoms of inattention and hyperactivity/impulsivity in children with ADHD (13). However, other studies have reported that PS exerts limited effects on hyperactivity/impulsivity or overall ADHD symptoms (14). In addition, PS has demonstrated efficacy in addressing internalizing and externalizing behavioral problems in children with ADHD, such as emotional dysregulation and behavioral impulsivity (15). Moreover, phosphatidylserine (PS) has been evaluated in several randomized placebo-controlled studies, which consistently indicated certain clinical therapeutic effects in improving attention, impulsivity, and behavioral regulation among children with ADHD (13, 15, 16). These findings provide supportive evidence for the therapeutic potential of PS in improving ADHD-related symptoms. Nevertheless, research on the effects of PS on core ADHD symptoms remains limited and warrants further investigation.

This study employed an open, randomized trial design to compare the effectiveness of PS and atomoxetine in improving core ADHD symptoms as well as internalizing and externalizing behavioral problems. This study aimed to assess whether PS can serve as an effective adjunctive treatment for ADHD, achieving therapeutic outcomes comparable to those of atomoxetine.

## 2 Methods

### 2.1 Study design

This study adopted an open-label controlled design and was conducted among children aged 6–13 years diagnosed with ADHD. Neither participants nor outcome assessors were blinded to group allocation. From March to June 2023, 60 children with ADHD were recruited from the Child and Adolescent Mental Health Clinic at Hangzhou Normal University Affiliated Hospital. Participants were allocated to the PS group (n = 30) and the atomoxetine group (n = 30) using a simple randomization procedure. Group allocation was based on a computer-generated random number sequence and was performed by an independent researcher who was not involved in outcome assessment. Sample size estimation was conducted based on scale-based outcome measures. According to previous research, an effect size of 0.48 was assumed, with a significance level (α) of 0.05 and a statistical power of 0.80 (15). Using G*Power software, the estimated minimum sample size required was 18 participants per intervention group. Considering an anticipated dropout rate of approximately 20% during the intervention period, 30 participants were ultimately recruited for each intervention group. This study was approved by the Ethics Committee of the Affiliated Hospital of Hangzhou Normal University (Ethics Number: 2025E2-HS-033).

### 2.2 Inclusion and exclusion criteria

Inclusion criteria required that participants (1) were interviewed and diagnosed with ADHD according to DSM-5 criteria by a qualified psychiatrist; (2) were aged between 6 and 13 years; (3) had a Raven’s Standard Progressive Matrices score > 80; and (4) had received no ADHD-related medication use within the last month.

Exclusion criteria were (1) a history of autism spectrum disorder, mood disorders, or tic disorders; (2) intellectual functioning below the normal range (Raven’s Standard Progressive Matrices score < 80); and (3) a history of traumatic brain injury or neurological disorders (e.g., epilepsy, cerebral palsy) or the presence of other severe physical diseases.

### 2.3 Study procedure

Participants were divided into two groups, each receiving a different treatment regimen. The PS group received oral phosphatidylserine (Zhili100, produced by Beijing Fukesi Bio-Technology Co., Ltd.) at a dose of 100 mg twice daily. The atomoxetine group received oral administration of atomoxetine at a dose based on the child’s body weight. The initial dose was 0.5 mg/kg, which was gradually increased weekly until a target daily dose of 1.2 mg/kg was reached. Both groups received continuous treatment for three months. To reduce potential confounding effects, participants were required to be free of psychotropic medication for at least one month prior to enrollment. During the study, potential confounding factors, including concomitant medication use and behavioral interventions, were systematically recorded and monitored through regular follow-up visits.

### 2.4 Efficacy measures

ADHD symptoms: The SNAP-IV (Swanson, Nolan, and Pelham-IV Parent Form) scale was used to evaluate core ADHD symptoms. The scale uses a 0–3-point scoring system across 26 items, including inattention (items 1–9), hyperactivity/impulsivity (items 10–18), and oppositional defiant disorder (items 19–26). The total score reflects symptom severity. Clinical research has shown that this scale has high reliability and validity (17, 18).

Internalizing and externalizing problems: The Achenbach Child Behavior Checklist (CBCL) was used to assess internalizing and externalizing problems in children with ADHD (19). The scale consists of two parts: social competence and behavioral problems. The behavioral problems section includes 113 items and assesses internalizing problems (e.g., withdrawal, somatic complaints, anxiety, and depression) and externalizing problems (e.g., rule-breaking behavior and aggressive behavior).

### 2.5 Statistical methods

Data analysis was performed using SPSS 22.0 software. Results are expressed as mean ± standard deviation (SD). The chi-square test was used to analyze gender differences between the two groups. Independent samples t-tests were used to evaluate differences in age and baseline scale scores. Analysis of Covariance (ANCOVA) was used to compare changes in SNAP-IV and CBCL scale scores between the PS and atomoxetine groups before and after treatment, with baseline scores included as covariates. Paired t-tests were performed within each treatment group to examine pre–post differences in scale scores. All hypotheses were two-tailed, and p < 0.05 was considered statistically significant. The results were adjusted using the Bonferroni correction for multiple comparisons.

## 3 Results

### 3.1 Data overview

A total of 60 children were initially enrolled in the study. During the intervention period, two children in the PS group dropped out due to noncompliance with daily supplementation, and two children in the atomoxetine group withdrew because of missed follow-up visits. Therefore, data from 56 participants were included in the final analysis, with 28 children in each group. Analyses were conducted using complete-case data, and no imputation for missing data was performed.

The PS group consisted of 28 children (22 boys and 6 girls, aged 6–12 years), and the atomoxetine group also consisted of 28 children (24 boys and 4 girls, aged 6–13 years).There were no statistically significant differences between the two groups in gender, age, or baseline scores on the SNAP-IV and CBCL (p > 0.05), except for a marginal difference in CBCL internalizing behavior scores (p = 0.06). Therefore, an analysis of covariance (ANCOVA) was conducted to control for this potential baseline difference. Detailed baseline data are presented in **Table 1**.

**Table 1:** Demographic and clinical baseline characteristics of the cohort(mean ± SD).

Characteristic	PS(n=28)		Atomoxetine(n=28)		t	*p*
	Mean	SD	Mean	SD		
Demographic characteristic						
Males, n(%)	22(78.6)		24(85.7)		χ2 = 0.49	0.49
Age	8.68	1.85	9.36	1.37	1.56	0.12
SNIP-IV						
Hyperactive/Impulsive	0.77	0.56	1.03	0.64	1.60	0.12
Oppositional Defiant Disorder	0.70	0.55	0.92	0.61	1.46	0.15
Inattention	1.43	0.64	1.59	0.65	0.92	0.36
Total	25.36	12.50	30.89	13.19	1.61	0.11
CBCL Parent						
Internalizing Problems	6.71	4.70	9.04	3.98	1.93	0.06
Externalizing Problems	12.29	6.22	14.57	7.54	1.18	0.25

P values are based on two-sided t-test for independent samples.

### 3.2 Comparison of SNAP-IV parent rating scale scores before and after intervention

**Figure 1:**
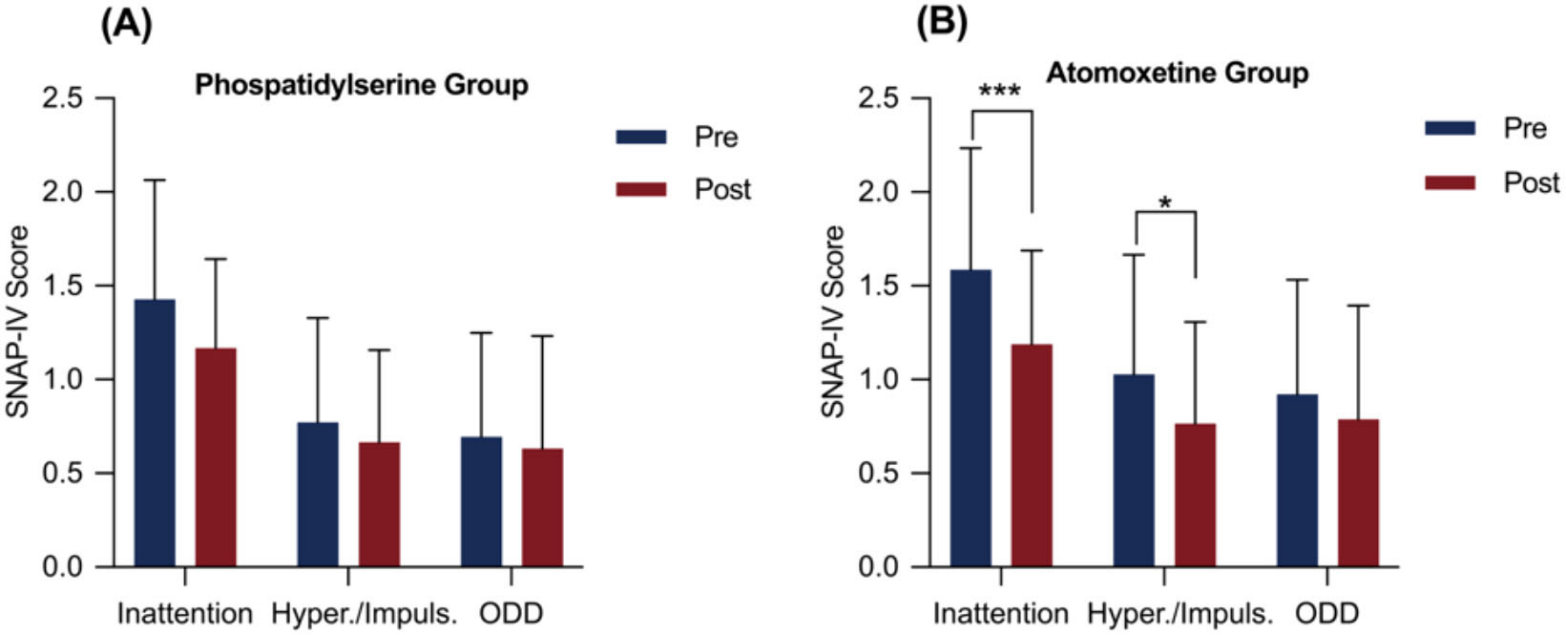
SNAP-IV scores before and after the intervention in the phosphatidylserine and atomoxetine groups. Error bars represent standard deviation. *p < 0.05, ***p < 0.001 after Bonferroni correction.

The effects of PS and atomoxetine treatment on the SNAP-IV components are shown in **Table 2**. In the PS group, no significant differences were observed in SNAP-IV scores before and after the intervention (attention deficit: M = 0.26, SD = 0.60, Bonferroni-corrected p > 0.05, Cohen’s d = 0.43, 95% CI = [0.04, 0.82]; hyperactivity/impulsivity: M = 0.11, SD = 0.46, corrected p > 0.05, Cohen’s d = 0.23, 95% CI = [-0.15, 0.60]; total: M = 3.82, SD = 10.27, corrected p > 0.05, Cohen’s d = 0.37, 95% CI = [-0.01, 0.75]; **Figure 1A**). However, in the atomoxetine group, significant reductions were observed in attention deficit (M = 0.40, SD = 0.50, corrected p < 0.05, Cohen’s d = 0.80, 95% CI = [0.36, 1.22]), hyperactivity/impulsivity (M = 0.26, SD = 0.49, corrected p < 0.05, Cohen’s d = 0.54, 95% CI = [0.13, 0.93]), and total scores (M = 7.00, SD = 9.38, corrected p < 0.05, Cohen’s d = 0.75, 95% CI = [0.32, 1.16]) after treatment compared with baseline (**Figure 1B**).

**Table 2:** SNAP- IV scores at baseline and endpoint in the PS and Atomoxetine groups (mean ± SD).

SNAP-IV Subscale	PS		T	*p*	Atomoxetine		t	*p*
	Baseline	Month-3			Baseline	Month-3		
Inattention	1.43 ± 0.64	1.17 ± 0.48	2.30	0.03	1.59 ± 0.65	1.19 ± 0.50	4.21	0***
Hyperactivity/Impulsivity	0.77 ± 0.56	0.67 ± 0.49	1.22	0.23	1.03 ± 0.64	0.77 ± 0.54	2.83	0.009*
Oppositional Defiant Disorder	0.70 ± 0.55	0.63 ± 0.60	0.68	0.50	0.92 ± 0.61	0.79 ± 0.61	1.34	0.10
Total	25.36 ± 12.50	21.54 ± 10.81	1.97	0.06	30.90 ± 13.19	23.89 ± 11.72	3.95	0.001**

P values are based on paired sample t-test. *p < 0.05, **p < 0.01, ***p < 0.001 after Bonferroni correction.

### 3.3 Comparison of CBCL scores before and after intervention

**Figure 2:**
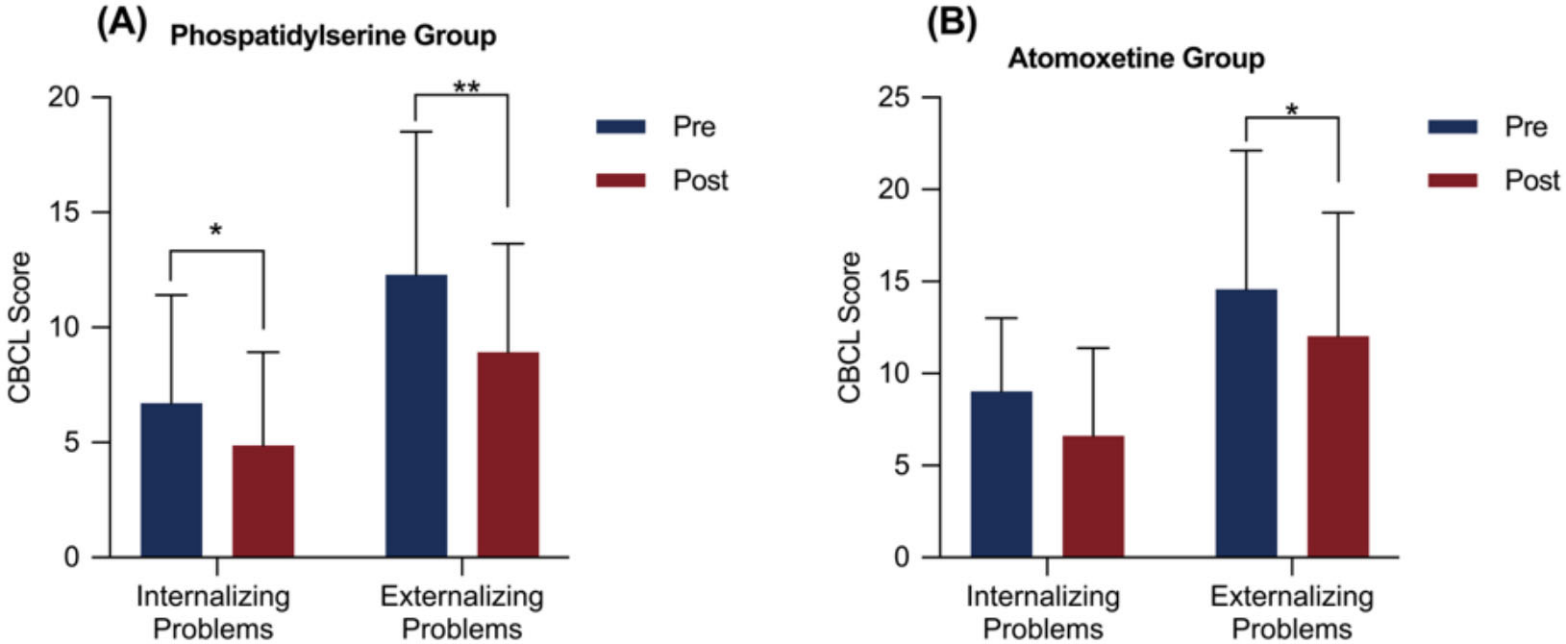
CBCL scores before and after the intervention of the phosphatidylserine and atomoxetine groups. Error bars represent standard deviation. *p < 0.05,**p < 0.01 after Bonferroni correction.

**Figure 3:**
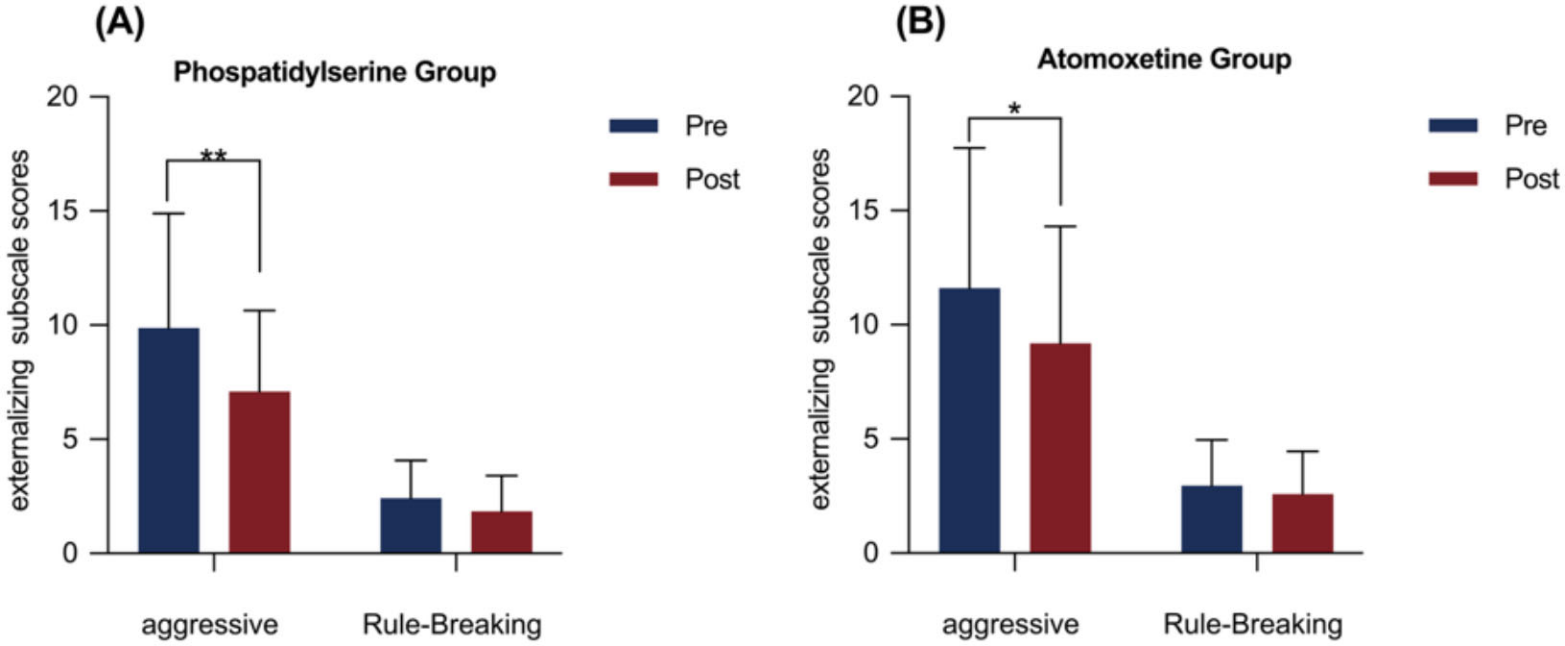
Externalizing behavior subscale scores before and after the intervention in the phosphatidylserine and Atomoxetine groups. The graph displays scores for both the aggressive behavior and rule-breaking behavior subscales. Error bars represent standard deviation. *p < 0.05, **p < 0.01 after Bonferroni correction.

The effects of PS and atomoxetine treatment on CBCL components are shown in **Table 3**. In the PS group, significant reductions were observed in both internalizing and externalizing behavior scores after the intervention (internalizing: M = 1.83, SD = 3.28, corrected p < 0.05, Cohen’s d = 0.56, 95% CI = [0.12, 0.99]; externalizing: M = 3.38, SD = 5.20, corrected p < 0.05, Cohen’s d = 0.65, 95% CI = [0.20, 1.1]; **Figure 2A**). In the atomoxetine group, only the externalizing behavior score significantly decreased after treatment (M = 2.54, SD = 5.42, corrected p < 0.05, Cohen’s d = 0.47, 95% CI = [0.07, 0.86]; **Figure 2B**). Further analyses of internalizing and externalizing subdomains revealed that both groups exhibited significant reductions in aggressive behaviors after treatment (PS: M = 2.79, SD = 4.24, corrected p < 0.05, Cohen’s d = 0.66, 95% CI = [0.21, 1.1]; atomoxetine: M = 2.43, SD = 4.90, corrected p < 0.05, Cohen’s d = 0.50, 95% CI = [0.10, 0.88]; **Table 4**, **Figure 3**).

**Table 3:** CBCL scores at baseline and endpoint in the PS and Atomoxetine groups(mean ± SD).

CBCL Subscale	PS		t	*p*	Atomoxetine		t	*p*
	Baseline	Month-3			Baseline	Month-3		
Internalizing Problems	6.71 ± 4.70	4.88 ± 4.06	2.74	0.01^*^	9.04 ± 3.98	6.61 ± 4.79	2.26	0.03
Externalizing Problems	12.29 ± 6.22	8.92 ± 4.72	3.18	0.004^**^	14.57 ± 7.54	12.04 ± 6.70	2.48	0.02^*^

P values are based on paired t-test. *p < 0.05, **p < 0.01 after Bonferroni correction.

**Table 4:** Externalizing subscale scores at baseline and endpoint in the PS and atomoxetine groups(mean ± SD).

Externalizing Subscale	PS		T	*p*	Atomoxetine		t	*p*
	Baseline	Month-3			Baseline	Month-3		
aggressive	9.88 ± 5.02	7.08 ± 3.56	3.22	0.004^**^	11.61 ± 6.14	9.18 ± 5.11	2.62	0.01^*^
Rule-Breaking	1.67 ± 0.34	1.58 ± 0.32	1.69	0.11	2.96 ± 1.99	2.57 ± 1.87	1.55	0.13

P values are based on paired t-test. *p < 0.05, **p < 0.01 after Bonferroni correction.

### 3.4 Comparison of SNAP-IV and CBCL scores between groups

The atomoxetine group exhibited greater reductions in all SNAP-IV dimensions and total scores than the PS group, although the differences were not statistically significant (p > 0.05).

For CBCL externalizing behavior scores, the PS group showed a greater reduction than the atomoxetine group, but the difference was also not statistically significant (p > 0.05).

### 3.5 Safety and adverse events

No serious adverse events were observed during the study period. In the atomoxetine group, gastrointestinal discomfort was reported in 28 participants, and sleep-related symptoms such as somnolence and night awakening were reported in 14 participants. In contrast, no adverse events were reported in the PS group, indicating good tolerability and safety of PS supplementation in children with ADHD.

## 4 Discussion

This study examined the associations between phosphatidylserine (PS) supplementation and the core symptoms of ADHD, as well as internalizing and externalizing behavioral problems, in children with ADHD. The findings indicate that while PS did not produce significant reductions in core ADHD symptoms, it was associated with notable reductions in internalizing and externalizing behavioral problems, particularly aggressive behaviors.

Our findings suggest that children with ADHD who received atomoxetine exhibited significant improvements in attention deficit, hyperactivity/impulsivity, and total scores, which is consistent with previous studies (20, 21). In contrast, the association between PS and core symptoms was more limited. While several studies have suggested that PS may improve inattention and hyperactivity/impulsivity symptoms in children with ADHD (13, 16), other studies have indicated that PS does not affect hyperactivity/impulsivity or overall ADHD symptoms (5). In our study, PS did not show significant improvement as measured by the SNAP-IV subscales after Bonferroni correction. Future studies are warranted to further explore the potential role of PS in alleviating core ADHD symptoms.

Our study also found that children with ADHD who took PS showed significant improvements in internalizing and externalizing behavioral problems, particularly in aggressive behavior. This finding aligns with that of Manor et al., who demonstrated that PS was associated with improvements in emotional and behavioral problems in children with ADHD (15). In contrast, children receiving atomoxetine showed significant improvements only in externalizing behaviors, with no substantial changes in internalizing behaviors. This pattern is consistent with prior evidence suggesting that atomoxetine is associated with modest changes in behavioral problems, often with small effect sizes (20). It should be noted that at baseline, the atomoxetine group exhibited slightly higher internalizing problem scores than the PS group (p = 0.06). Although this difference did not reach statistical significance, it may still represent a potential confounding factor influencing behavioral outcomes. To minimize this potential bias, baseline internalizing scores were included as covariates in the ANCOVA model when comparing post-treatment effects. Nevertheless, residual confounding cannot be entirely excluded and should be considered when interpreting between-group differences. Overall, these results suggest that PS may be more strongly associated with emotional and behavioral regulation than with core ADHD symptoms and may therefore warrant investigation as an adjunctive therapeutic option.

This study has several limitations. First, the relatively small sample size may limit statistical power and reduce the generalizability of the findings to broader populations of children with ADHD. Second, although this study adopted a randomized design, the absence of a placebo control and blinding may have introduced expectancy effects and reporting bias, particularly for parent- or clinician-rated behavioral outcomes. These factors may have influenced the observed changes and should be considered when interpreting the results. Accordingly, the findings should be regarded as exploratory rather than confirmatory. In addition, this study relied exclusively on behavioral assessments and therefore could not directly examine the neurobiological mechanisms underlying the observed behavioral changes. Although previous studies have proposed potential neurobiological roles of phosphatidylserine (22, 23), such mechanisms were not assessed in the present study and remain to be investigated in future research. Future studies with larger sample sizes, placebo-controlled and blinded designs, as well as complementary neurobiological assessments, are needed to further evaluate these preliminary findings and to explore the potential role of PS in emotional and behavioral regulation in children with ADHD.

## 5 Conclusion

In conclusion, this study provides exploratory evidence suggesting that phosphatidylserine (PS) supplementation may be associated with improvements in certain behavioral problems in children with ADHD. While PS was not associated with significant changes in core ADHD symptoms, it was associated with reductions in internalizing and externalizing behavioral problems, particularly aggressive behavior. Given the exploratory nature and methodological limitations of the study, these findings should be interpreted cautiously. Larger, well-designed randomized controlled trials are required to further examine the potential role of PS in the management of behavioral difficulties in children with ADHD.

## Data Availability

The raw data supporting the conclusions of this article will be made available by the authors, without undue reservation.
